# Human milk microbiota in sub-acute lactational mastitis induces inflammation and undergoes changes in composition, diversity and load

**DOI:** 10.1038/s41598-020-74719-0

**Published:** 2020-10-28

**Authors:** Alba Boix-Amorós, Maria Teresa Hernández-Aguilar, Alejandro Artacho, Maria Carmen Collado, Alex Mira

**Affiliations:** 1 Department of Biotechnology, Spanish National Research Council (IATA-CSIC), Institute of Agrochemistry and Food Technology, Paterna, Spain; 2 Department of Health and Genomics, Center for Advanced Research in Public Health, FISABIO Foundation, Valencia, Spain; 3 Dr Peset Lactation Unit, National Health Service, Dr. Peset University Hospital, Valencia, Spain; 4 Present Address: Department of Genetics and Genomic Sciences, Icahn School of Medicine at Mount Sinai, 1470 Madison Avenue, New York, NY 10029 USA

**Keywords:** Clinical microbiology, Microbial communities, Microbial genetics

## Abstract

Sub-acute mastitis (SAM) is a prevalent disease among lactating women, being one of the main reasons for early weaning. Although the etiology and diagnosis of acute mastitis (AM) is well established, little is known about the underlying mechanisms causing SAM. We collected human milk samples from healthy and SAM-suffering mothers, during the course of mastitis and after symptoms disappeared. Total (DNA-based) and active (RNA-based) microbiota were analysed by 16S rRNA gene sequencing and qPCR. Furthermore, mammary epithelial cell lines were exposed to milk pellets, and levels of the pro-inflammatory interleukin IL8 were measured. Bacterial load was significantly higher in the mastitis samples and decreased after clinical symptoms disappeared. Bacterial diversity was lower in SAM milk samples, and differences in bacterial composition and activity were also found. Contrary to AM, the same bacterial species were found in samples from healthy and SAM mothers, although at different proportions, indicating a dysbiotic ecological shift. Finally, mammary epithelial cell exposure to SAM milk pellets showed an over-production of IL8. Our work therefore supports that SAM has a bacterial origin, with increased bacterial loads, reduced diversity and altered composition, which partly recovered after treatment, suggesting a polymicrobial and variable etiology.

## Introduction

Human milk is a complex and live fluid, containing a relatively diverse and potential beneficial microbiota under healthy conditions^1^, which enhances gut microbiota colonization, likely stimulates commensal tolerance and supports the maturation of the immune system^2–5^. Occasionally, the lactating mother is afflicted with the development of mastitis, which frequently arises during the first 6 weeks post-partum and is one of the main causes of early weaning^6–8^. According to the World Health Organization (WHO), mastitis affects up to 33% of lactating women^9^, but this is likely biased by the difficulties for defining the disease. Classically, mastitis is defined as an inflammation of the breast, accompanied of infection or not^6,9,10^. Most researchers consider that lactational mastitis has an infectious origin. Symptoms appear when a blockage of the milk ducts occur, presumably due to the overgrowth of some bacterial species which form biofilms and trigger inflammation^11–14^. According to its course, lactational mastitis can be classified into different types, being acute (AM) and sub-acute mastitis (SAM) the most prevalent among breastfeeding women. AM can be easily identified due to the intensity of its symptoms, namely erythema, pain, swelling, fever and other general symptoms. *Staphylococcus aureus* is considered the main causative agent in AM, producing toxins responsible for the systemic symptoms^12,15–17^. SAM, albeit courses with milder symptoms, is most prevalent among lactating women and therefore represents one of the principal causes of undesired cessation of lactation. Based on bacterial cultures from human milk samples, *Staphylococcus epidermidis* has been proposed as the predominant species responsible of SAM^18–20^, as well as other coagulase negative staphylococci (CNS) and viridans streptococci^12,21^. These are frequently encountered in healthy skin microbiota and human milk, and can occasionally overgrow and form thick biofilms in the milk ducts^22,23^, leading to milk stasis and opportunistic infections, which result in the symptoms of SAM previously described^11,24^ Identifying SAM can be challenging, and a poor diagnostic and/or treatment can lead to recurrent or chronic infections. Microbial culture is the standard diagnose procedure, but this technique is time-consuming, and can result in false negatives (as many microbial species cannot be grown under standard laboratory conditions). In addition, most bacteria associated with lactational mastitis can be frequently detected in healthy mother’s milk, which further complicates the diagnosis^15^. Studies addressing the microbiology of SAM are limited, and information about bacterial loads during human lactational mastitis is scarce. In addition, most human milk microbiota studies based on molecular techniques focus on the total bacterial DNA composition, not considering that part of its DNA may correspond to dead or inactive bacteria, as well as free bacterial DNA. For this reason, RNA-based sequencing of human samples is being used to clarify the elusive aetiology of some diseases with complex microbial origin^25,26^.

The aim of the current work was to describe the human milk bacterial composition in mothers suffering SAM, taking into account total and active bacteria (as inferred by DNA and RNA 16S rRNA gene sequencing, respectively), and loads, in order to better characterize the etiology of the disease and find potential bacterial biomarkers. In addition, bacterial pellets from human milk were exposed to a mammary epithelial cell line, in order to investigate their potential role in inflammatory processes.

## Results

### Study population

Fifty-one women were enrolled in the study, including 24 healthy-controls, 24 SAM and 3 AM. From the whole data set, some drop-outs occurred during the study (one mother from the control group, and 5 mothers from the SAM group abandoned the study before collecting the second sample). Characteristics of mothers and infants are summarized in Table 1.

**Table 1:** Study population’s information.

	Healthy	Sub-acute mastitis	Acute mastitis	Total/average
Study population, n	24	24	3	51
Maternal age, years ± SD	34.83 ± 2.85	35.08 ± 5.32	34.33 ± 2.08	34.92 ± 4.12
Days post-partum (t0 sample)	45.66 ± 2.89	44.83 ± 25.13	41.66 ± 43.41	45.04 ± 23.46
t1 sample*	10.83 ± 3.16	17 ± 5.08	14.33 ± 4.5	16.65 ± 5.09
Weight-gain during pregnancy ± SD	14.83 ± 13.31	13.71 ± 4.22	11.00 ± 3.46	14.08 ± 9.54
Vaginal delivery	19/24	17/24	2/3	38/51
Maternal antibiotics during study	0/24	14/24	3/3	17/51
Infant age (days) ± SD	45.66 ± 2.89	44.83 ± 25.13	41.66 ± 43.41	45.04 ± 23.46
Infant antibiotics during study	0/24	0/24	0/3	0/51

*In mothers with mastitis it corresponds to recovery time.

### Total and active bacterial load increase in human milk during mastitis

**Figure 1:**
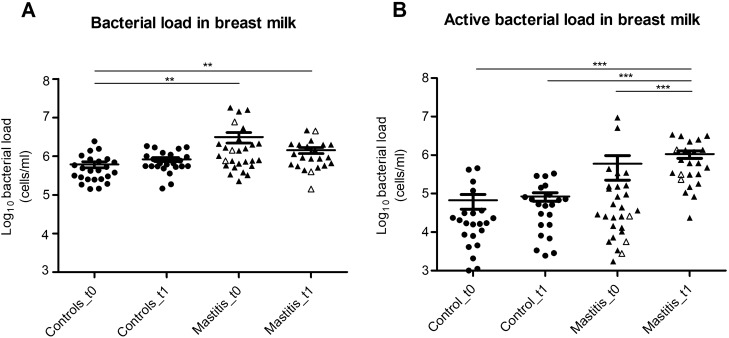
Bacterial load in human milk of healthy mothers and mothers suffering lactational mastitis. Plots show means with standard errors. (**a**) Bacterial load, as inferred from qPCR of the 16S rRNA gene of the bacterial DNA. (**b**) Active bacterial load, as inferred from qPCR of the 16S rRNA gene of the bacterial RNA (cDNA). Controls_t0, (n = 24); Controls_t1, (n = 23); Mastitis_t0, (SAM, n = 24; AM, n = 3); Mastitis_t1, (SAM, n = 19; AM, n = 3). t0, samples collected during the course of mastitis symptoms, or first sample collected in healthy controls; t1, samples collected after the clinical symptoms disappeared, or samples collected from healthy controls one week after the first sample collection. Acute Mastitis samples are represented with white triangles in the graph. **p < 0.01 and ***p < 0.001, non-parametric Kruskal Wallis test. Created in GraphPad Prism 5 v5.04 (www.graphpad.com).

Quantification of the 16S rRNA gene through qPCR of both DNA (total bacterial load) and cDNA (active bacterial load) showed significantly increased bacterial loads in the mastitis samples during the course of the symptoms (Fig. 1). Mean total bacterial load in the control samples was 610,127 cells/ml (SEM = 110,218) and 828,850 cells/ml (SEM = 100,692) at first and second time point, respectively. Mean total load in the mastitis samples (including SAM and AM) during the course of the symptoms reached 3,137,000 cells/ml (SEM = 956,632), which was significantly higher as compared to controls at the same time point (non-parametric Kruskal Wallis test, *p* < 0.01). After the symptoms had disappeared, mean total load in the mastitis group decreased to 1,430,000 cells/ml (SEM = 259,037), although the values were still significantly higher as compared to controls at time 0, and thus, bacterial load did not fully return to healthy levels at this time point (non-parametric Kruskal–Wallis test, *p* < 0.01). Mean active bacterial load was significantly lower as compared to total bacterial load in all groups (non-parametric Kruskal–Wallis test, *p* < 0.001), except in the mastitis group at time 1. Similar values were observed in the control samples at the two studied time points (time 0 = 67,064 cells/ml [SEM = 27,505]; and time 1 = 84,808 cells/ml [SEM = 21,117]). Mean active bacterial load increased in the mastitis group during the course of symptoms, up to 598,395 cells/ml (SEM = 373,253) although this difference was not significant, perhaps as a consequence of the larger data variation, which could be affected by RNA instability. Mean active load in the mastitis group after symptoms disappeared increased up to 1,601,000 cells/ml (SEM = 229,296), and this difference was significant when compared to all the other groups (non-parametric Kruskal–Wallis test, *p* < 0.001).

### Bacterial richness and diversity are reduced during lactational mastitis

**Figure 2:**
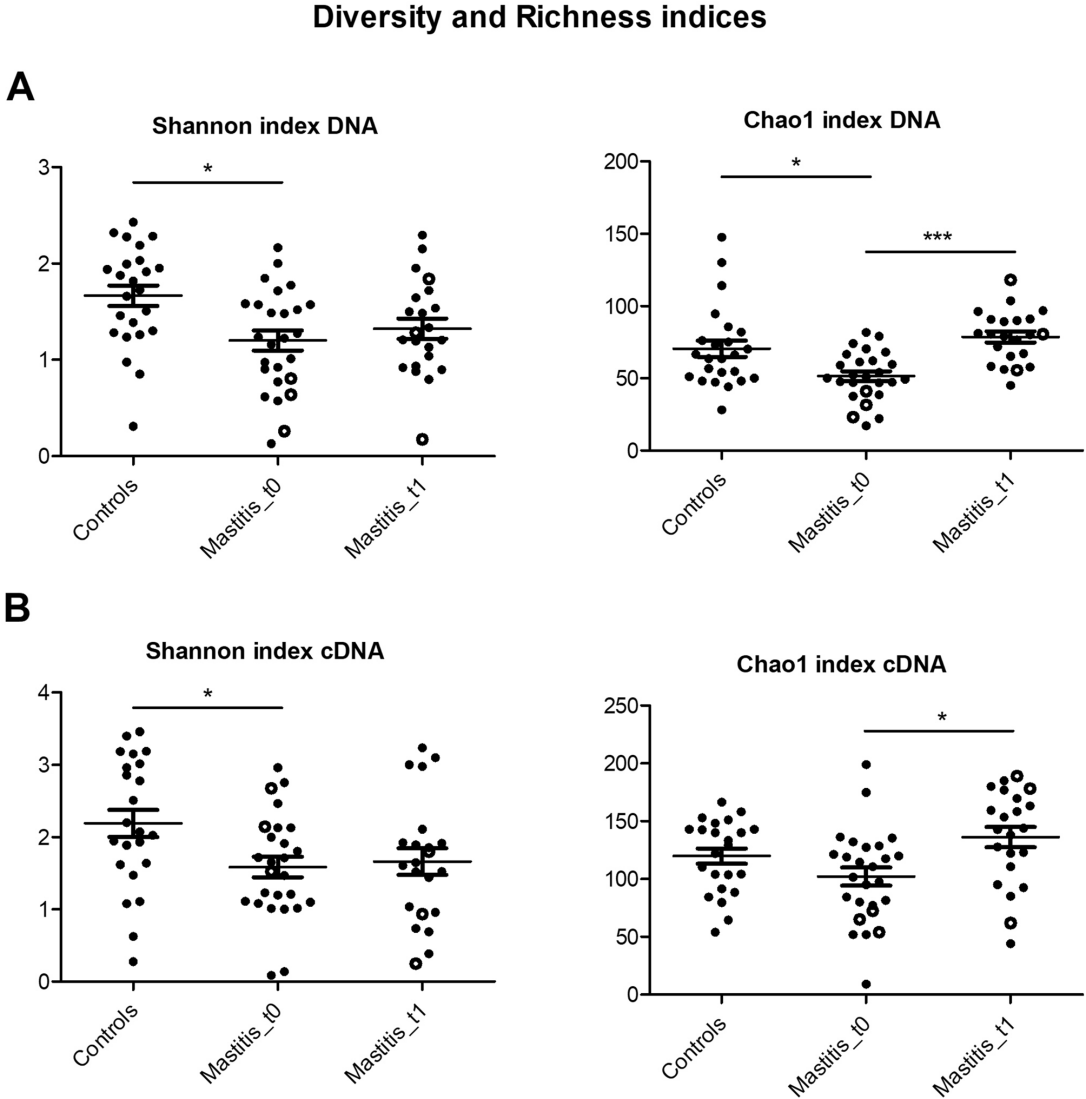
Bacterial diversity and richness in human milk samples from healthy and mastitis-suffering women. Plots show human milk microbiota genus-level diversity and richness (here presented with Shannon and Chao1 indices), with means and standard errors. (**a**) Represents microbiota DNA Shannon and Chao1 indices. Controls, n = 24; Mastitis_t0, n = 26; Mastitis_t1, n = 22. (**b**) Shows microbiota RNA Shannon and Chao1 indices. Controls, n = 23; Mastitis_t0, n = 26; Mastitis_t1, n = 22. Acute mastitis samples are represented with white circles, n = 3. t0 = samples during the course of the symptoms; t1 = samples after symptoms disappeared. *p < 0.05 and ***p < 0.001, non-parametric Kruskal Wallis test. Created in GraphPad Prism 5 v5.04 (www.graphpad.com).

After sequencing, one DNA sample (SAM group, time 0) and two cDNA samples (control group and SAM group, time 0) were not considered for further analyses due to the small number of sequences yielded. Total bacterial DNA diversity (as measured by the Shannon Index) and richness (as measured by the Chao1 Index) were lower during the course of mastitis symptoms (Fig. 2a; non-parametric Kruskal–Wallis test, *p* < 0.05). Diversity levels did not return to control levels after the symptoms had disappeared, although the estimated bacterial richness significantly increased (non-parametric Kruskal–Wallis test, *p* < 0.001). A similar pattern was observed at the RNA level: Active bacterial diversity was lower during the mastitis symptoms (Fig. 2b; non-parametric Kruskal–Wallis test, *p* < 0.05), and the bacterial richness in the RNA group increased after symptoms had disappeared (non-parametric Kruskal–Wallis test, *p* < 0.05). Although there was a trend towards lower richness in the mastitis group during the symptoms, it did not reach statistical significance.

### Total and active bacterial composition change during and after the course of sub-acute mastitis

**Figure 3:**
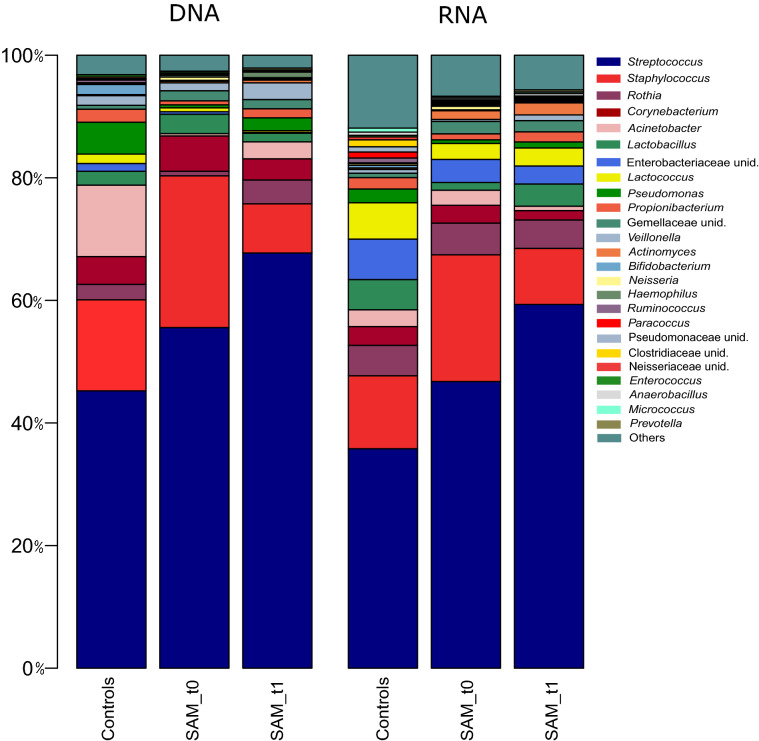
Bacterial composition in human milk samples of healthy mothers and mothers suffering sub-acute mastitis. The bar plot shows average percentage of abundance of the genus-level bacteria (DNA) and active bacteria (RNA) detected in the human milk samples by means of Illumina MiSeq sequencing of the 16S rRNA gene. Controls: DNA, n = 24; RNA, n = 23; SAM_t0: DNA, n = 23; RNA, n = 23; SAM_t1; DNA, n = 19; RNA, n = 19. t0 = samples during the course of the symptoms; t1 = samples after symptoms disappeared. Due to the small sample size (n = 3), acute mastitis samples were not included in the bar plots. Created in R software v 3.5.1 (2018–07-02) (https://www.R-project.org).

**Figure 4:**
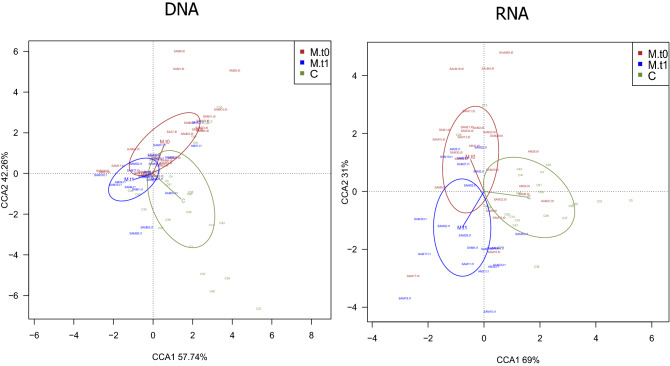
Human milk microbiota patterns in healthy mothers and mothers suffering lactational mastitis. Constrained correspondence analyses (CCA), which here emphasize variations in microbiota OTU-level patterns, in human milk from healthy mothers and mothers suffering lactational mastitis at two time points, at DNA-level (*p* = 0.015), and active microbial RNA-level (*p* = 0.04). The percentage of variation explained by constrained correspondence components is indicated on the axes. t0 = samples during the course of the symptoms; t1 = samples after symptoms cessation. p-values for CCA plots were determined by Adonis, and indicate if health status and/or time can significantly explain data variability. Controls (C): DNA, n = 24; RNA = 23; Mastitis t0: DNA, n = 26; RNA = 26; Mastitis t1: DNA, n = 22; RNA, n = 22. Created in R software v 3.5.1 (2018–07-02) (https://www.R-project.org).

*Streptococcus* and *Staphylococcus* were the two most abundant bacterial genera, both in the DNA (67.12% and 8.00%, respectively) and RNA sequences (51.23% and 14.57%, respectively) (Fig. 3). No statistically significant effect of maternal antibiotics intake, maternal and infant age, delivery mode nor maternal weight gain during pregnancy, were detected on human milk microbial composition (*p* > 0.05, MaAsLin test). At genus level, SAM samples at time 0 had lower levels of *Pseudomonas* than healthy controls (unpaired Wilcoxon test, adjusted p-value = 0.003), and lower levels of *Acinetobacter* than SAM at time 1 (paired Wilcoxon test, adjusted p-value = 0.031). When looking at the active (RNA-based) bacterial composition, Firmicutes phylum was higher in the SAM group at time 0, as compared to controls (Wilcoxon test, p-value = 0.01), at the expense of a depletion in Proteobacteria (Wilcoxon test, p-value = 0.001) and to SAM at time 1 (Wilcoxon test, p-value = 0.05). *Peptoniphilus, Prevotella*, and *Finegoldia* were at higher levels in the active portion of SAM at time 1 as compared to healthy controls (adjusted p-value = 0.0087, 0.019 and 0.042, respectively), while healthy controls were enriched in *Neisseria* (adjusted p-value = 0.042), suggesting that the bacterial composition was not fully recovered after the clinical symptoms had disappeared. Relative abundances of bacterial genera per group are summarized in Table S1. At OTU (species) level, the impact of health status on human milk microbiota was also reflected in differences in bacterial composition between healthy controls, mastitis during the course of symptoms (time 0) and mastitis after symptoms cessation (time 1), both at DNA level (Adonis p-value = 0.015, CCA analysis), and active RNA level (Adonis p-value = 0.04, CCA analysis) (Fig. 4). As inferred from DNA analysis, controls appeared more dispersed in the CCA plot, while mastitis groups were more similar in composition and clustered closer to each other. At RNA level, although there was also some overlap, the three groups clustered separately, and the highest divergence was explained by axis 1, which separates controls from mastitis groups. Thus, the CCA plots also support a different bacterial composition at the species level in mastitis and control groups, with a partial recovery after the symptoms disappeared. *Streptococcus mitis/oralis, Streptococcus salivarius, Acinetobacter johnsonii, Streptococcus lactarius* and *Rothia mucilaginosa* were the most abundant species detected in the human milk samples at DNA level (Table S2). *Streptococcus mitis/oralis, Streptococcus salivarius, Staphylococcus epidermidis, Rothia mucilaginosa* and *Streptococcus lactarius* were the most abundant active bacteria detected (RNA level) (Table S3)*. Staphylococcus aureus* was more abundant in SAM, both at time 0 (adjusted *p-*value = 0.001), and time 1 (adjusted *p-*value = 0.0003, Wilcoxon test), as compared to healthy controls. *Porphyromonas endodontalis* (adjusted *p*-value = 0.003) and *Streptococcus peroris* (adjusted *p-*value = 0.003) were more prevalent in SAM group at time 1, as compared to controls. Paired Wilcoxon test showed that *Acinetobacter johnsonii* was more abundant in SAM group at time 1 as compared to SAM at time 0 (adjusted *p-*value = 0.025). *Staphylococcus aureus* and *Streptococcus lactarius* were also more active in SAM samples both at time 0 (unpaired Wilxocon test, adjusted *p-*value = 0.023; *p* = 0.013, respectively) and time 1 (unpaired Wilxocon test, adjusted *p-*value = 0.005; *P* = 0.018, respectively), as compared to controls. *Streptococcus peroris* was significantly more active in SAM group at time 1, as compared to controls (unpaired Wilxocon test, adjusted *p-*value = 0.006) and to SAM time 0 samples (paired Wilcoxon test, adjusted *p-*value = 0.012).

**Figure 5:**
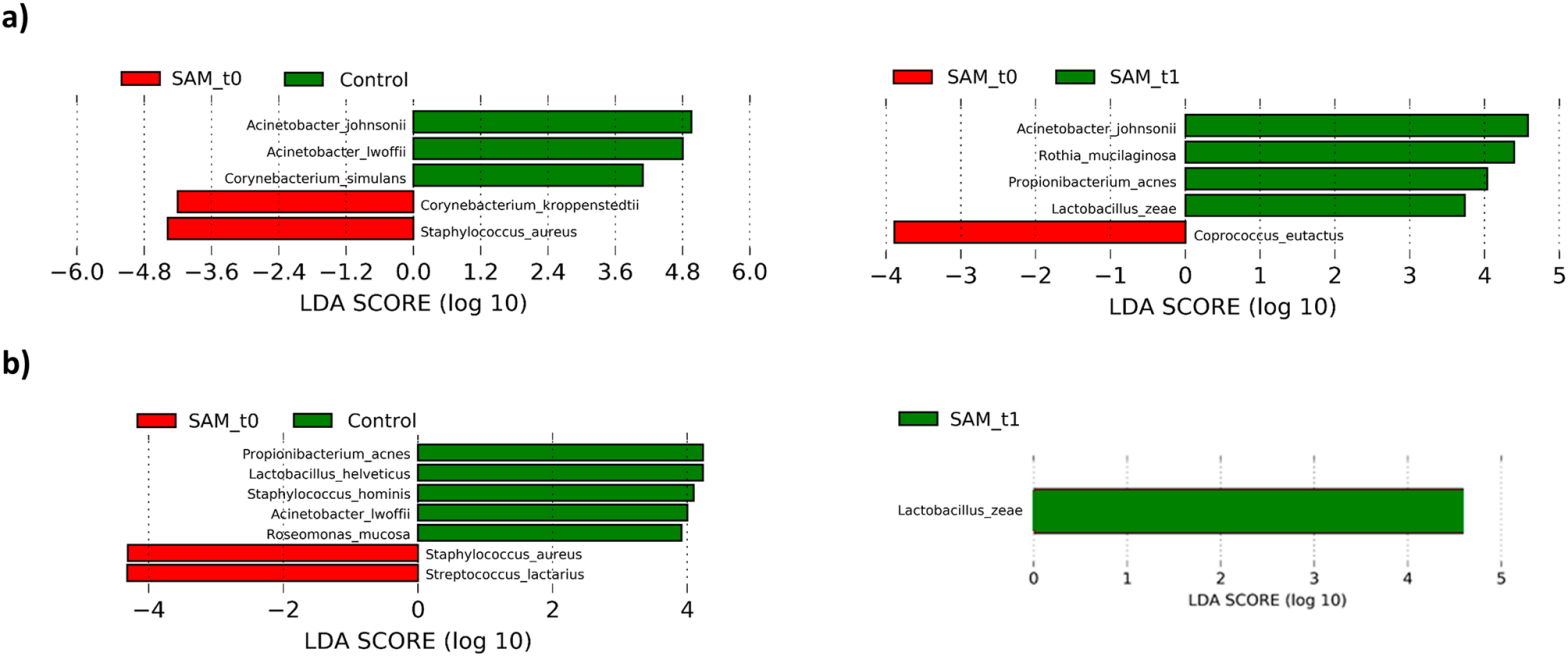
Human milk bacterial OTUs associated with sub-acute mastitis. The plots show differentially abundant bacteria between healthy controls and SAM during symptoms, and between SAM during and after symptoms had disappeared, as inferred from: (**a**) DNA (controls, n = 24; SAM_t0, n = 23; SAM_t1, n = 19); and (**b**) RNA (controls, n = 23; SAM_t0 = 23; SAM_t1 = 19). The LEfSe algorithm was used for biomarker discovery; the threshold for logarithmic discriminant analysis (LDA) score was 2, and *p* < 0.05. Created in GraphPad Prism 5 v5.04 (www.graphpad.com).

In addition, LEfSe algorithm was applied in order to further examine potential biomarkers of SAM disease (Fig. 5). Bacteria at significantly higher levels in healthy mother’s milk, as compared with mothers suffering from SAM included *Acinetobacter johnsonii, Pseudomonas viridiflava*, *Corynebacterium simulans*, *Paracoccus marcusii*, *Pseudomonas fragi* and *Acinetobacter lwoffii.* Conversely, *Corynebacterium kroppenstedtii*, *Staphylococcus aureus*, and *Prevotella nanceiensis* were observed in increased abundance in human milk during SAM. Significant differences in bacterial abundance were also observed when analysing the active bacterial fraction of the samples (Fig. 5b).

**Figure 6:**
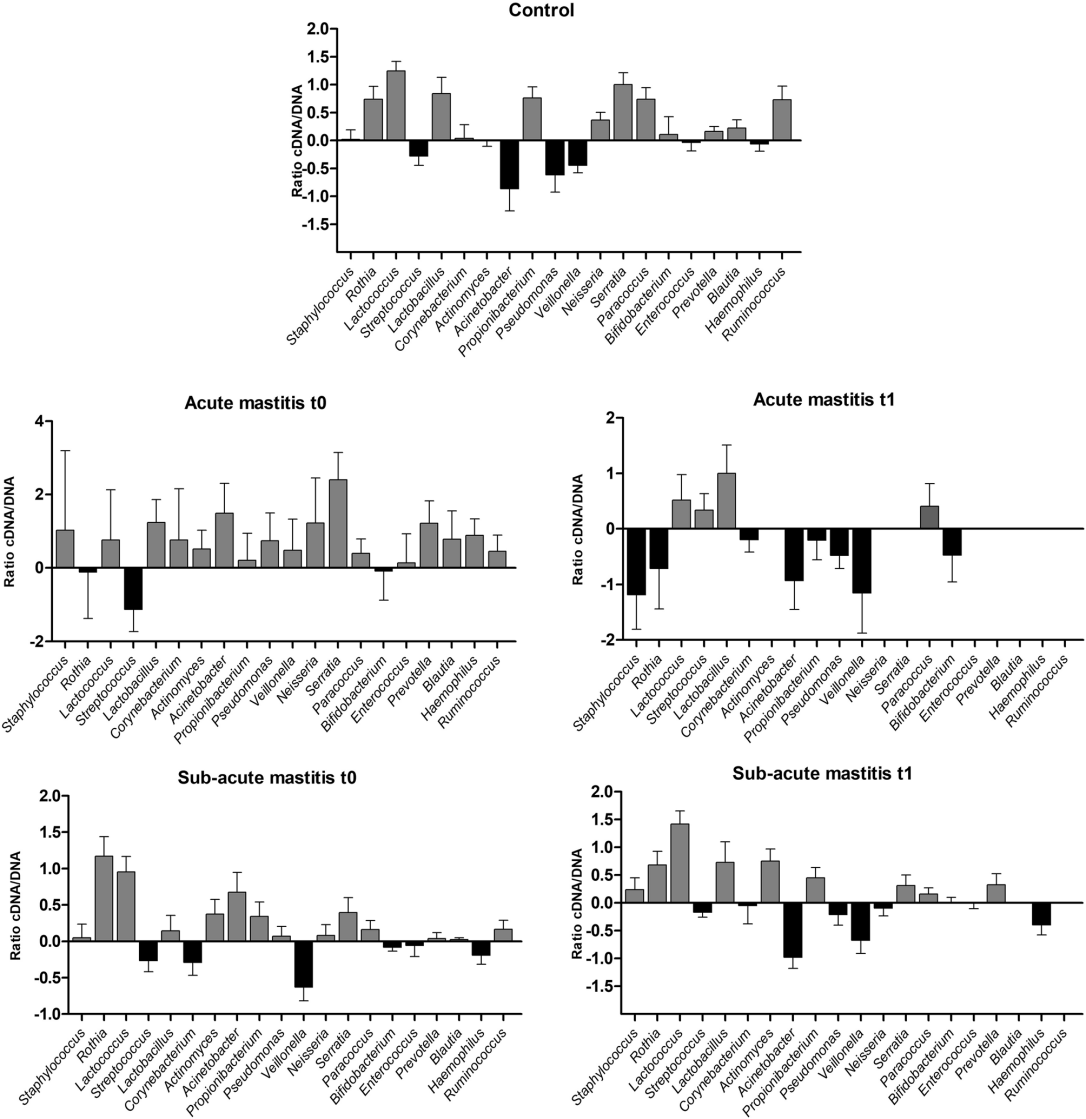
Average bacterial activity indices in human milk in health and during mastitis. The graph shows average bacterial activity indices, corresponding to the ratio between the proportion of each bacterial genus in the RNA- and DNA-based sequences, and the standard error of the mean (SEM) per groups. Controls, n = 23; Sub-acute mastitis t0, n = 23; Sub-acute mastitis t1, n = 19. Positive ratios are represented in grey, negative ratios in black. Created in GraphPad Prism 5 v5.04 (www.graphpad.com).

Under the assumption that pathogens involved in mastitis should be over-represented in the RNA samples relative to their levels in the DNA, an analysis of the bacterial “Activity Index”, corresponding to the ratio between the proportion of each bacteria in the RNA-based and DNA-based sequences, was performed. The average activity indexes for the different genera in each group are shown in Fig. 6. The data show that, in acute mastitis, there is an increase in the activity of *Staphylococcus*, which decreases (negative index values) after the symptoms subsided. *Streptococcus*, on the other hand, has a negative activity index during acute mastitis, whereas it shifts to positive activity values when the symptoms disappeared. In SAM samples, only a few patterns can be observed, like an increased activity during mastitis and a decreased activity after recovery.

**Figure 7:**
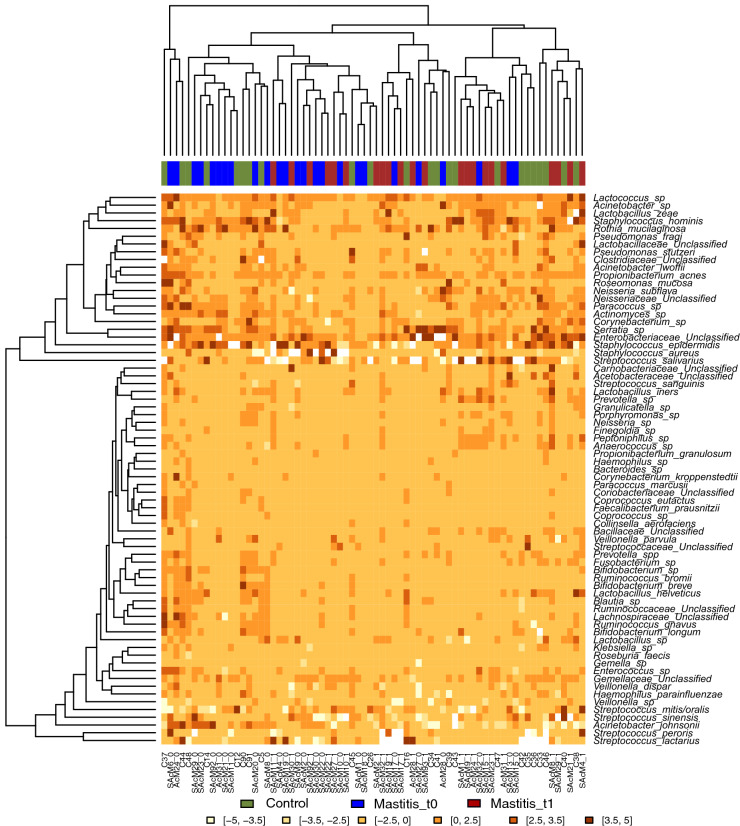
Bacterial activity and cluster analysis in human milk in health and mastitis. The heatmap shows the activity indices corresponding to the ratio between the proportion of each bacterial species in the RNA-based and DNA-based sequences. Controls, n = 23; SAM_t0, n = 23; SAM_t1, n = 19. Indices are represented in different colours depending of their higher or lower values in the samples. Created with R software v 3.5.1 (2018-07-02) (https://www.R-project.org).

In order to further study potential associations between bacterial activity and mastitis, the activity indices of bacterial species in each samples was compared (Fig. 7). Although some milk samples clustered according to health status, results showed a large overlap in bacterial activity ratios between healthy mothers and those with mastitis before and after treatment. This is in agreement with the above-mentioned biomarker discovery analysis in that the etiology of SAM is complex and probably polymicrobial. In addition, the presence of some bacteria at low activity under health conditions that increase in activity during mastitis suggests that some etiological agents of the disease may already be present in health, and that disease onset may be the outcome of a microbial dysbiosis, whose triggering factors should be identified.

### Human milk bacteria exposure to mammary epithelial cells and release of IL8

**Figure 8:**
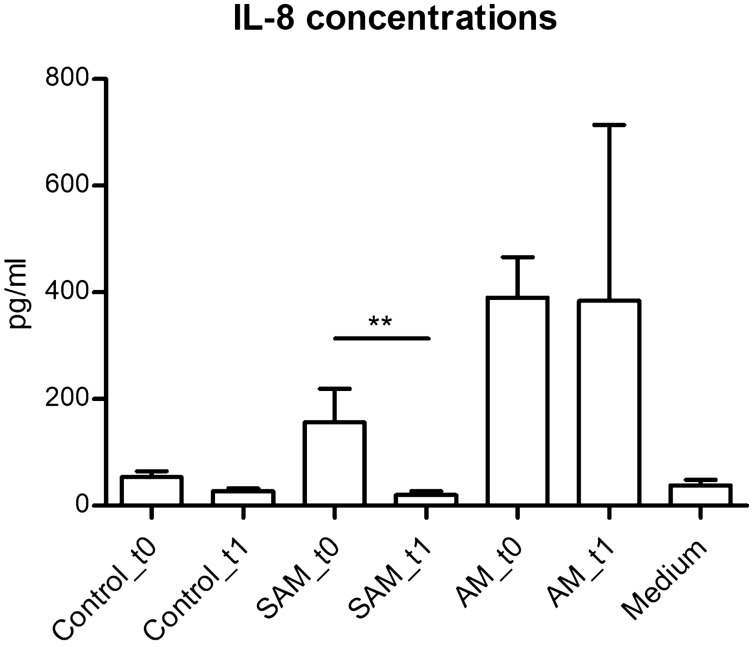
Levels if IL8 produced by mammary cells after exposure to bacteria in human milk. The bar plots show levels of IL8 released by cells from a mammary epithelial cell line when exposed to bacterial pellet from healthy controls (Controls_t0, n = 18; Controls_t1, n = 22); sub-acute mastitis (SAM_t0, n = 21; SAM_t1, n = 17); and acute mastitis (AM_t0, n = 2; AM_t1, n = 3). Supernatants from mammary cells exposed only to culture medium were used as negative controls (n = 8). **p < 0.01, non-parametric Kruskal–Wallis test. Created in GraphPad Prism 5 v5.04 (www.graphpad.com).

To study the potential pro-inflammatory effect of bacteria associated to SAM, human milk pellets were co-incubated with a mammary epithelial cell line for 24 h. Results showed higher levels of IL8 production in cells exposed to pellets from SAM during the course of the symptoms, which significantly decreased after symptoms disappeared (non-parametric Kruskal–Wallis test, *p* < 0.01) (Fig. 8). As expected, levels of IL8 from cell supernatants exposed to AM pellets were higher during and after the symptoms, as compared to the other groups, although the small sample size did not allow statistical analyses. Although we did not find any correlation between absolute bacterial loads and the levels of IL8 in the samples, we observed a positive correlation between the genus *Staphylococcus’* relative abundance and the levels of IL8 in the DNA group (Spearman’s *ρ*, 0.363, *p* value, 0.003). This positive correlation was also observed at the cDNA level, although it was not statistically significant (Spearman’s ρ, 0.229, *p* value, 0.007).

## Discussion

Sub-acute mastitis is a fastidious and common disease among lactating mothers, representing one of the main causes of undesired weaning. Despite its high prevalence and impact on maternal-infant health, SAM is undervalued and under-diagnosed^12^. Studies performed on human milk during lactational mastitis up to date point to an altered bacterial profile, increased abundance in opportunistic pathogens and lower bacterial diversity^14,16,18,20^. Classically, AM has been associated to *Staphylococcus aureus* infections^12,15–17^. SAM, on the other hand, has been associated with increased *Staphylococcus epidermidis* presence^18–20^, and with lower abundances of other coagulase negative staphylococci (CNS), and viridans streptococci^21^*.* Most of these results, however, were derived from culture-dependent analyses, which are known to be biased by false negative rates. Only two studies, up to date, used culture-independent methods to analyse human milk microbial profiles during mastitis^18,20^. In both studies, lower bacterial diversities in mastitis samples were reported, in addition to enrichment in opportunistic pathogens.

In the present study, both total (DNA-based) and active (RNA-based) bacterial composition and load of the SAM and healthy human milk samples have been studied. Results show that total bacterial load was significantly higher during the time of mastitis symptoms, and decreased after the symptoms ceased, although loads remained significantly higher as compared to healthy controls. Interestingly, active load after the symptoms’ cessation was significantly higher as compared to healthy controls and mastitis during the symptoms. This could indicate increased bacterial activity levels during the process of re-balancing in the bacterial community. After sequencing the 16S rRNA gene amplicons, we observed lower bacterial diversities both at DNA and RNA level during mastitis, in agreement with previously reported data^18,20^. Richness was also lower during the disease and increased after symptoms had disappeared. However, diversity was not recovered to control levels after the symptoms disappeared. Given that samples were collected short after the symptoms had disappeared (1–4 days after mother’s stopped suffering the symptoms was considered as inclusion criteria), data suggest that a full recovery of bacterial communities takes longer than the remission of clinical symptoms and future studies should collect samples at a later stage. Decreased microbial diversity and/or richness associated to microbial dysbiosis have been previously described in several disease conditions, such as inflammatory bowel disease^27,28^, colorectal cancer^29^, tooth decay^30^ or celiac disease^31^, among others.

Activity indices, calculated as the ratio between the proportion of each microorganism in the cDNA and the DNA samples from each donor, show differentially active bacteria in the milk from healthy and mastitis-suffering donors. However, milk samples did not cluster according to bacterial activity and maternal health status (controls vs SAM), as observed in the CCA results, and this overlap supports a polymicrobial, variable and dysbiotic etiology of SAM. A plausible explanation for the absence of a unique, clear causal agent responsible of SAM etiology probably lies in the fact that many bacteria associated with SAM are commonly present in human milk under healthy conditions. Thus, contrary to what occurs in AM, SAM may not always be the outcome of an external, or environmental infection but may be caused by a dysbiosis in the pre-existing inhabitants of human milk. Dysbiosis has been introduced as a useful concept to explain disease etiology in cases where classical infection by an external pathogen cannot be identified^26^. In other inflammatory diseases where pathogenic communities are present under health conditions, an environmental factor, ranging from diet changes to immune alterations, could be the triggering factor leading to a dysbiosis^32–34^, and further studies should identify if any external factor could initiate an unbalance in milk microbial communities.

Despite the high inter-individual bacterial variability of human milk among samples, specific differences between groups were observed. During SAM, *Pseudomonas* and *Acinetobacter* were diminished, as compared to healthy controls and after symptoms disappeared (SAM, time 1), respectively. Interestingly, after cessation of the symptoms, SAM (time 1) samples were enriched in typical oral inhabitants, such as *Streptococcus* and *Porphyromonas* (DNA); and *Prevotella* (RNA). In addition, potential anaerobic and opportunistic pathogenic genera like *Finegoldia* and *Peptoniphilus* were also increased in SAM at time 1, which could reflect an imbalance in human milk after the mastitis episode. At OTU level, results showed that milk from mothers suffering SAM were enriched in *Staphylococcus aureus*, even after the symptoms had disappeared, and were also significantly more active as compared to healthy controls. Given that *S. aureus* was detected in only 1 out of 24 healthy mothers, its higher proportion and prevalence could have been due to an infection from an environmental source. In most other cases, however, the disease-associated bacteria were also found in milk from healthy mothers. *Streptococcus lactarius*, for instance, was also found to be significantly more active in SAM during and after symptoms cessation, but it was also found at high prevalence in healthy mothers (13 out of 23 samples). In those cases, it is therefore possible that a change in the proportion of pre-existing microorganisms could trigger the inflammatory process. In addition, after symptoms disappeared, samples were enriched in typical oral inhabitants such as *Porphyromonas endodontalis* and *Streptococcus peroris.* Given that oral health alterations, especially gingivitis and periodontitis, are very frequent in mothers during pregnancy and lactation^35,36^, the potential role of oral bacteria in triggering inflammation in the mammary tissue should be considered and tested in future studies.

LEfSe biomarker discovery analyses showed several OTUs associated to health, including *Acinetobacter johnsonii, Corynebacterium simulans*, and *Acinetobacter lwoffiii,* among others*. Corynebacterium kroppenstedtii*, *S. aureus* and oral species such as *Prevotella nanceiensis* were among the identified SAM biomarkers. *Corynebacterium kroppenstedtii* has been previously isolated from granulomatous mastitis and breast abscesses samples^37,38^. Others like *Acinetobacter johnsonii*, *Propionibacterium acnes, Lactobacillus helveticus* and *Lactobacillus zeae* were significantly more abundant after symptoms had disappeared. Interestingly, *P. acnes* is a predominant bacterium in the skin microbiome, which has shown anti-*S. aureus* activity in vitro^39,40^. Thus, future studies should focus not only on potentially pathogenic organisms, but also on bacteria which could potentially contribute to health conditions, as promoting their growth with pre- or probiotics could prove to be a strategy to prevent or treat SAM^11,41^.

RNA analysis showed that active *Lactobacillus iners*, *Neisseria subflava*, *Streptococcus lactarius*, *Streptococcus cristatus* and *S. aureus* were associated with SAM during the symtoms; while others such as *P. acnes*, *Staphylococcus hominis*, *Acinetobacter lwoffii* and *Lactobacillus helveticus* were associated with health. Some of the potential pathogens deserve further study. *Lactobacillus inners*, for instance, is a member of the vaginal normal microbiota^42,43^, although it has been associated to vaginal dysbiosis^44^. This species can produce a toxin, named as innerlysin, which may cause cell damage^45^. Thus, its potential role in SAM should be tested in the future. In addition, our results show that pellets from SAM milk samples, containing bacteria, induce pro-inflammatory IL8 release by mammary epithelial cells, supporting an infectious origin of SAM. Although we did not observe an overall correlation between bacterial load and IL8 release in the cells, our results showed a specific correlation between the genus *Staphylococcus* and IL8 production (only statistically significant at the DNA level), further supporting the potential pro-inflammatory role of this genus.

From a methodological point of view, we propose that the RNA:DNA activity index could be a useful approach to detect pathogenic species in complex human samples, as pathogens would be expected to have higher activity at the disease site than commensal inhabitants.

In summary, our data support a bacterial origin of SAM, with a polymicrobial and variable aetiology, probably the result of a dysbiosis in the milk microbial population.

## Materials and methods

### Subjects and sampling

A total of 51 mothers participated in the study. Among them, 24 presented symptoms of SAM, 3 presented symptoms of AM and were included for comparison, and the remaining 24 were completely healthy. Human milk samples were collected between 9 and 90 days after delivery, at two time points: during the course of the symptoms (time 0) and after the symptoms cessation (time 1) in the mastitis group; and during a medical consultation to the doctor (time 0), and a second visit a week after (time 1) in the control group. Details of pregnancy and delivery, mother/infant health status, medicines consumption, lactation, and clinical symptoms during mastitis were collected at recruitment through a detailed questionnaire. Mothers (over 18 years old) were recruited at the Breastfeeding Unit of Dr. Peset Hospital and at the Alfafar Health Center (Valencia, Spain). Women were considered to have SAM when presenting breast pain (usually described as profound, needle-like and/or burning pain) accompanied or not by lumps in the breast tissue and without general symptoms^18^. Women were considered to have AM when presenting profound pain in the breast accompanied by at least two of the following symptoms: local inflammation signs (breast redness, local hyperthermia, or sensitive lump), fever and general discomfort^18^. Controls were healthy breastfeeding woman who did not present any of the previous symptoms. Exclusion criteria were: suffering from immunological, metabolic or other severe diseases; or having received antibiotics or probiotics 15 days prior to first sample donation. Breastfeeding counselling was offered to all mothers suffering from breast pain. After any other cause of breast pain (such as an incorrect lactating posture, or infant’s short frenulum) was discarded and the diagnosis of sub-acute mastitis was confirmed, mothers were instructed on breastfeeding massage techniques and optimal lactation positioning. Treatments with anti-inflammatory drugs and/or probiotics were prescribed. In fourteen cases from the SAM group, symptoms persisted after 7 days and antibiotics were prescribed. Mothers suffering AM received antibiotics and anti-inflammatory drugs, following the recommended standards. Prior sampling, nipples and mammary areola were cleaned with chlorhexidine soap and sterile water and rinsed with sterile saline solution. After manually discarding the first milk drops, samples were collected with a Medela Symphony breast-pump (Medela, Baar, Switzerland) in sterile collection units. Samples were collected in the morning, at least 1 h after the last feeding. Written, informed consent was obtained from all participants at the time of sample collection. Protocol was approved by the Ethical Committee of Clinical Research from the Dr. Peset University Hospital (Valencia, Spain), with reference number CEIC 19/16. All research was performed in accordance with the relevant guidelines and regulations.

### Sample processing and DNA/RNA isolation

Human milk samples (4 ml) were centrifuged at 13,000×*g* for 10 min at 4 °C, discarding fat and whey. Total DNA and RNA were isolated from pellets by using the MasterPure Complete DNA & RNA Purification Kit (Epicentre, Madison WI, USA) as previously described^46^. A mix of 150–212 μm and 425–600 μm acid washed glass beads (Sigma-Aldrich) were added and samples were put through two cycles of vigorous mixing (6.5 m/s) for 60 s with 60 s rest period between runs, in a FastPrep-24 5G Instrument (MP Biomedicals, Santa Ana, CA, USA). Nucleic acids were eluted in 30 μl TE buffer. 10 μl of each nucleic acids’ suspension were transferred to a new nuclease-free tube, and treated with the DNA-free DNA Removal Kit (Invitrogen, Carlsbad, CA, USA) to remove DNA and keep only RNA. 0.1 volume of the 10X DNase I Buffer and 1 µl of rDNAse I were added to the tubes, and incubated at 37 °C for 30 min. This step was repeated a total of three times to ensure complete removal of DNA. 0.1 volume of the DNAse Inactivation Reagent was added, incubated for 2 min at RT and centrifuged at 10,000×*g* and 4 °C for 2 min. Supernatants containing clean RNA were transferred to new Eppendorf tubes. DNA and RNA concentrations were measured in a Nanodrop Spectrophotometer (ThermoScientific).

### cDNA synthesis

cDNA was synthesized from RNA by using the Transcriptor First Strand cDNA Synthesis Kit (Roche Life Science, Basel, Switzerland). A mix of: 1 µg of each RNA sample, 1 µl of Anchored-oligo (dT) primer, (2.5 μM); 2 µl of Random Hexamer Primer (60 μM) in a final volume of 13 µl. The primer-template mix was heated at 65 °C for 10 min. 4 µl of Transcriptor Reverse Transcriptase Reaction Buffer (1 × 8 mM MgCl_2_); 0.5 µl of Protector RNase Inhibitor (20U), 2 µl of Deoxynucleotide Mix (1 mM each) and 0.5 μl (10 U) of the Transcriptor Reverse Transcriptase (final volume: 20 µl) were added to each tube. Tubes were gently mixed and incubated for 10 min at 25 °C, followed by 30 min at 55 °C, and 5 min at 85 °C. Final cDNA products were stored at − 80 °C.

### Detection of 16S rRNA gene by qPCR

Total bacterial load (DNA-based) and active bacterial load (cDNA-based) of the samples were analysed through quantitative PCR (qPCR) amplification and detection of the 16S ribosomal RNA gene. Each reaction mixture of 10 μl was composed of: 5 μl of Light Cycler 480 SYBR Green I Master mix (Roche Life Science), 0.25 μl of each specific primer (concentration 10 μM) and 1 μl of template. Amplifications were performed in a Light Cycler 480 Real-Time PCR System (Roche Life Science), using the following conditions: 95 °C for 5 min, (95 °C for 10 s, 60 °C for 20 s, 72 °C for 20 s) (40 cycles), followed by dissociation curve analysis. All amplifications were performed in duplicates and negative controls were included in each qPCR plate. In all, 5 qPCR plates were used for the analyses of all the samples. Primers sequences were as follows: F—5ʹ-CGTGCCAGCAGCCGCGG-3ʹ and R—5ʹ-TGGACTACCAGGGTATCTAATCCTG-3′^47^. Cq values in each sample were transformed in bacterial cell numbers per ml of milk by comparison with a standard curve obtained with flow cytometry. This standard was generated by using DNA extracted from 10 million bacterial cells from 10 pure cultures of different species commonly found in human milk (*Streptococcus epidermidis* CECT 231, *Bifidobacterium dentium* DSM 20436, *Acinetobacter lwoffii* CECT 453, *Corynebacterium matruchotii* DSMZ 20635, *Lactobacillus casei* (lab’s isolate), *Lactobacillus acidophilus* CECT 4179, *S.aureus* strain 240*, Pseudomonas aeruginosa* ATCC 15442, *Rothia mucilaginosa* (lab’s isolate) and *Streptococcus mitis* DSMZ 12643). Bacterial cells were quantified and sorted using a BD FACSAria II cytometer (BD, East Rutherford, NJ, USA). DNA from all species were extracted, pooled and diluted in serial ten-fold dilutions to create a single standard curve. Samples that showed Cq values higher than the negative control were considered negative for bacterial detection.

### Bacterial composition and active bacterial composition of human milk samples

A total of 75 human milk samples were analysed through sequencing of the 16S rRNA gene. Controls, time 0 (n = 24); Mastitis, time 0 (n = 25; 22 SAM and 3 AM); Mastitis, time 1 (n = 23; 20 SAM, 3 AM). Controls at time 1 were not included in further steps. Prior to sequencing, DNA and cDNA were pre-amplified by using universal bacterial degenerate primers 27F—5ʹ-AGAGTTTGATCMTGGCTCAG-3ʹ and 926R—5ʹ-CCGTCAATTCMTTTRAGT3ʹ, which comprise the hypervariable regions V1–V5 of the gene. This step was performed by using the high-fidelity ABGene DNA polymerase (ThermoScientific, Waltham, Mass., USA) with an annealing temperature of 52 °C and 10 cycles, in order to minimize amplification biases that could arise with longer cycles^48^. PCR products were purified using Nucleofast 96 PCR filter plates (Macherey–Nagel, Düren, Germany), and concentrations were measured with a Qubit 3 Fluorometer (ThermoScientific). An Illumina amplicon library was performed following the 16S rRNA gene Metagenomic Sequencing Library Preparation Illumina protocol (Part #15044223 Rev. A). The primer sequences used target the 16S rRNA gene V3-V4 regions, resulting in a single amplicon of approximately 460 bp^49^. After amplification of the 16S rRNA gene, DNA and cDNA were sequenced in an Illumina MiSeq platform according to manufacturer’s instructions (Illumina) using the 2 × 300 bp paired-end protocol, at the FISABIO Institute (Valencia, Spain). No-template controls (NTCs) and negative controls during DNA extraction were included to rule out potential contaminations at the time of DNA extraction or sequencing.

### Data analysis and statistics

A quality assessment of the sequences was carried out using the PRINSEQ program^50^. Sequences were end-trimmed in 20 bp sliding windows, and those with average quality value < 30, and length < 250 bp were not considered for further analyses. Reads were pair-end joined using FLASH program applying default parameters^51^. Only overlapping paired-end reads were used for further analysis. The negative controls within the sequencing process produced 615 and 348 reads. The DNA and RNA extraction negative controls yielded 1995 and 1293 reads, respectively. *Agrobacterium*, Brucellaceae and Bradyrhizobiaceae were identified as contaminants and removed from the analyzed datasets^52^. Operational taxonomic units (OTUs) were generated by clustering reads at 97% of similarity by using VSEARCH^53^. Centroids (representative OTUs) were taxonomically classified at phylum, class, family, genus and species level using feature-classifier command of QIIME2^53^ version 2017.8 with Greengenes database (version gg_13_5). Sequences belonging to *Streptococcus* and *Staphylococcus* genera, whose 16S gene is highly similar among species, were clustered into OTUs at 100% similarity and > 400 bp alignment length by BLASTn analysis^54^, against a manually curated database for these genera, obtained from RDP Hierarchy Browser^55^. *Streptococcus mitis* and *Streptococcus oralis* were identical in the sequenced region and could not be distinguished from each other.

α-diversity analysis (Shannon and Chao1 indices), were calculated to estimate sample’s diversity and richness; and β-diversity (Bray Curtis dissimilarity index), to quantify the compositional dissimilarity between groups at OTU and genus level, using the R-package vegan^56^.

Canonical correspondence analysis (CCA) was performed by R software vegan package. In order to control the potential effects of maternal antibiotics intake, maternal age and days postpartum, MaAsLin multivariate analysis with linear model^57^ was applied. Adonis statistic for permutational multivariate analysis was used to measure differences in variance between groups, and Wilcoxon test implemented in R software was applied to determine significantly different bacterial genera between groups.

Bacterial-OTUs biomarker discovery was performed by linear discriminant analysis effect size (LEfSe) implemented on Galaxy^58^, in order to detect differentially abundant OTUs characterizing the populations of healthy and mastitis-suffering women. Other analyses and graphs were performed in GraphPad Prism 5 v5.04.

In all samples, a bacterial ‘Activity Index’ was calculated as the ratio between the proportion of each microorganism in the cDNA and the DNA samples from each donor. This ratio would allow the identification of those bacteria whose activity is higher or lower than expected based on their presence, as inferred by sequencing of the 16S rRNA gene. Data were then log-transformed so bacteria which were over- or under-represented in the RNA relative to the DNA fraction of each individual would be assigned positive or negative values of the Activity Index, respectively. Activity Indexes were represented with R in a heatmap, grouped by health status (Controls, SAM and AM) and time (t0, t1), eliminating bacteria at a proportion < 0.1 in more than 90% of the samples.

### Exposure of milk bacteria to a mammary gland epithelium cell line

The mammary epithelial cell line MCF7 (ATCC HTB-22), was seeded onto 96-well plates (30,000 viable cells per well) in complete growth medium (DMEM high glucose (Gibco, ThermoScientific) supplemented with 10% v/v inactivated fetal bovine serum (Sigma), 1 mM sodium pyruvate (Gibco), 0.1 mM non-essential amino acids (Gibco), 10 mM HEPES (Gibco), 2 mM l-glutamine (Gibco) and antibiotics (100 U/ml penicillin, 100 g/ml streptomycin (Gibco)). The cells were grown at 37 °C and 5% CO_2_ in an incubator for 2 days, and the integrity of the cell culture was checked with an inverted microscope. The medium was replaced with 200 μl fresh complete growth medium without antibiotics, containing the bacterial pellet from 500 μl of centrifuged milk samples. A total of 18 healthy controls, 21 SAM and 2 AM milk samples obtained at time 0; and 22 controls, 17 SAM and 3 AM milk samples obtained at time 1, were analysed in duplicates in the same experiment. Negative controls consisted of MCF7 cells incubated without bacteria. Co-incubation of mammary gland MCF7 epithelial cells with human milk bacteria was maintained for 24 h at 37 °C and 5% CO2 in an incubator. After co-incubation, culture supernatants were aspirated from wells and kept at 4 °C for measuring human IL8 concentration by ELISA (Invitrogen) using 25 uL of supernatants, following the manufacturer’s instructions.

## Supplementary information

Supplementary Legends.

Supplementary Tables.

## Data Availability

The datasets generated and analysed during the current study are available in the European Nucleotide Archive (ENA) repository, (“https://www.ebi.ac.uk/ena”). Sequence and meta-data are accessible under the study identifier PRJEB34421, and samples were deposited under the accession numbers: ERS3744372-ERS3744511.
