# Supplementary material for: Human milk microbiota in sub-acute lactational mastitis induces inflammation and undergoes changes in composition, diversity and load
Source: Sci Rep. 2020 Oct 28;10:18521. doi: 10.1038/s41598-020-74719-0 (PMC7595153; doi:10.1038/s41598-020-74719-0)
Supplement: Supplementary file 1 — Supplementary Legends. [file 41598_2020_74719_MOESM1_ESM.docx]

**Human milk microbiota in sub-acute lactational mastitis induces inflammation and undergoes changes in composition, diversity and load**

Alba Boix-Amorós^a,b*^, Maria Teresa Hernández-Aguilar^c^, Alejandro Artacho^b^, Maria Carmen Collado^a¶^, Alex Mira^b¶#^

^a^Institute of Agrochemistry and Food Technology; Spanish National Research Council (IATA-CSIC); Department of Biotechnology, Paterna, Spain.

^b^Department of Health and Genomics, Center for Advanced Research in Public Health, FISABIO Foundation, Valencia, Spain.

^c^Dr Peset Lactation Unit, Dr. Peset University Hospital, National Health Service, Valencia, Spain.

^#^Corresponding author

Email: [mira_ale@gva.es](mailto:mira_ale@gva.es) (AM)

^¶^These authors contributed equally to this work

*Present address: Department of Genetics and Genomic Sciences, Icahn School of Medicine at Mount Sinai. 1470 Madison Avenue. New York, NY 10029, USA

**Table S1. Mean relative abundances of bacterial genera per group.**

Controls_DNA, n=24; Controls_cDNA, n=23; SAM_t0_DNA, n=23; SAM_t1_DNA, n=23; SAM_t0_cDNA, n=19; SAM_t1_cDNA, n=19. t0= samples during the course of the symptoms; t1= samples after symptoms disappeared.

**Table S2. Relative abundances of bacterial species per sample in the DNA fraction**

Controls_DNA, n=24; Controls_cDNA, n=23; SAM_t0_DNA, n=23; SAM_t1_DNA, n=23; SAM_t0_cDNA, n=19; SAM_t1_cDNA, n=19; AM_t0_DNA, n=3; AM_t1_DNA, n=3; AM_t0_cDNA, n=3; AM_t1_cDNA, n=3. t0= samples during the course of the symptoms; t1= samples after symptoms disappeared.

**Table S3. Relative abundances of bacterial species per sample in the cDNA fraction**

Controls_DNA, n=24; Controls_cDNA, n=23; SAM_t0_DNA, n=23; SAM_t1_DNA, n=23; SAM_t0_cDNA, n=19; SAM_t1_cDNA, n=19; AM_t0_DNA, n=3; AM_t1_DNA, n=3; AM_t0_cDNA, n=3; AM_t1_cDNA
